# Discrimination Against Healthcare Workers by Patients and Colleagues, Affective Injustice and the Impact on Well‐Being and Practice

**DOI:** 10.1111/bioe.70115

**Published:** 2026-04-27

**Authors:** Brenda Bogaert

**Affiliations:** ^1^ Institute of Humanities in Medicine Lausanne University Hospital and University of Lausanne Lausanne Switzerland

## Abstract

Discrimination in healthcare is a pervasive issue that affects patients, healthcare providers, and quality of care. This article mobilizes the concept of affective injustice—a wrong done to someone as an affective being—to better understand the harms experienced by healthcare providers facing discrimination from both patients and colleagues. When patients discriminate, the duty to care often overrides the healthcare provider's ability to express justified emotions, particularly anger. In contrast, inter‐colleague discrimination frequently takes the form of microaggressions, which are difficult to challenge. Because of these difficulties, healthcare providers often feel pressured to engage in emotional regulation strategies to manage their own affects. While emotional regulation strategies may be helpful in the short‐term, we show that they do not address the root causes of discrimination and may exacerbate healthcare providers' distress, and impact care quality and staff retention. To better deal with these problems, the article will propose affective resources, opportunities, and freedoms that can be put into place to support healthcare providers and to make the workplace a more just space for all.

## 1 Introduction

While there is no consensus on the definition of discrimination, it is generally recognized that the term should be defined as treating individuals unfairly because of their race, color, religion, sex (including pregnancy, sexual orientation or transgender status), income, national origin, social status, disability, age, or genetic information. Discrimination is important in the clinical context, because it may affect healthcare quality and delivery [1]. Most research attention has focused on how *patients* experience discrimination and the negative consequences it entails, including poorer quality healthcare provision and conflictual provider‐patient relationships. [2, 3] Less attention has been given to what happens when healthcare providers experience discrimination in the workplace, whether from patients, colleagues, or management. Such discrimination is complex; it may involve interpersonal and collective dimensions, and it may be overt or subtle. Either way, it typically leads to emotional distress, anxiety, sadness, and frustration; however, it also has broader consequences for healthcare delivery, including reduced motivation at work and strained workplace relationships [4].

Bioethicists can and should develop a more robust approach to social justice, including creating a conceptual basis for addressing discrimination [5]. This article proposes to add to the conceptual debate by looking at the harms of discrimination related to well‐being. We will mobilize the concept of *affective injustice*—which may be defined as something done wrong to someone as an affective being [6]—to explore the affective harms that come from discrimination in the workplace. Affective injustice is a relatively new concept, having first been conceptualized in 2018 by Amia Srinivasan [7] to investigate the role of affects in social movements. Going against the grain, she shows that emotions like anger—typically viewed as negative—actually bring energy to social justice movements, helping to crystallize important issues at hand and bring others into the movement. However, given its negative reputation, expressing anger is often deemed inappropriate by others, including by the leaders of the social movements themselves. Activists are therefore encouraged to suppress their anger and to “calm down” to enable dialogue. Srinivasan describes these types of situations as *affective injustice*, situations in which a person must negotiate between apt emotional responses inspired by the injustice of their situation and the reception of these emotions by others. According to her, minimizing or hiding emotions in the face of an injustice often leads to a tragic dilemma, as it forces the person to choose between expressing their legitimate feelings or adapting to others' behavioral expectations of them (often by suppressing or hiding their anger). However, when the person is obliged to change their behavior to fit social expectations, it not only devalues what they feel, but it may also take away energy from the movement. From this perspective, affective injustice not only brings individual but also collective harms.

Since this first conceptualization of affective injustice, the concept has been brought closer to the epistemic injustice debate through the work of Archer and Mills [6]. These researchers define affective injustice as those situations of, “an injustice faced by someone specifically in their capacity as an affective being.” According to them, when one's affects are silenced or misunderstood, this may lead both to epistemic injustices but also to other harms related to well‐being. Indeed, if we can accept that affects are forms of knowledge and being in the world, the inability to express them, to be heard or understood, represents a wrong done to a person in their capacity as a knower [8]. However, it can also lead to other harms, in particular harms related to the person's well‐being, including feelings of isolation, confusion, additional stress, and anxiety. Concretely, this means that the concept of affective injustice is not just a side topic of epistemic injustice (although there are epistemic elements which should be taken into account), as it may also uncover specific problems related to affective harms.

Archer and Mills have also moved the concept forward by showing the link with emotional regulation studies in psychology, which is the practical means by which affective injustice happens in practice in the case of discrimination. Given that affects are devalued in certain situations, this creates explicit and implicit pressures to find ways to manage them, including their suppression. This is the domain of emotional regulation, first conceptualized by James Gross. He defines emotional regulation as, “the processes by which individuals influence which emotions they have, when they have them, and how they experience and express them” [9]. Emotional regulation is a learned capability, one that helps us to live with others and function in society; however, it can also go awry and cause harm to well‐being. They highlight in particular four emotional regulation strategies and their harms. These include: (1) *situational strategies*, which include managing the situations one finds oneself in, such as avoiding places and situations that make us feel a negative emotion. The problem with this strategy is that is does little to change the oppressive situation and often it is not possible to avoid one's oppressor (such as in the workplace). (2) *attentional strategies*, which involve managing what one attends to and shifting one's attention from the object that causes the emotion and directing one's focus elsewhere. These strategies often do little to change the situation or give adequate attention to what the person is feeling. (3) *cognitive strategies*, which involve managing how one appraises one's situation, such as deciding that we are wrong about a situation, or finding ways to understand the other's perspective. While these strategies may be helpful to develop a wider perspective, changing one's assessment of a situation may be harmful as it not only gives insufficient attention to one's experiences but can also put into doubt what the person knows. (4) *response modulation strategies*, which involve managing one's behavior and most significantly involves *emotional suppression strategies*, or active attempts to prevent expression of an emotion, such as trying to control one's facial muscles or voice.

As we can see from this list, while emotional regulation strategies are a social skill that helps us adapt to our environment and to live with others, they can go awry when they are based upon social pressures to conform to certain expectations and behaviors. This is because they do little to change the situation and can make the person feel even more isolated and distressed. In healthcare, emotional regulation strategies used by healthcare workers have been subject to an extensive literature in psychology. Most have shown that emotional regulation is a factor in resilience, enabling well‐being and helping to prevent burnout [10]. However, there has not been enough research attention to its negative effects, let alone when healthcare providers face discrimination. Indeed, due to the social and professional expectation that healthcare providers will not to show their emotions in order to be “good professionals,” healthcare workers in situations of discrimination are often put into a difficult situation: while they may be justifiably angry, frustrated, or even scared, they are expected to remain calm/unemotional. While this may help in certain situations of care work, emotional regulation strategies when dealing with discrimination may cause harm for the individual person and for the institution. Secondly, emotional regulation strategies for dealing with discrimination are different than some other situations due to their group‐based nature. Indeed, as Duker et al. [11] have shown, emotional regulation strategies are less amenable to repair due to group‐based discrimination as the individual person cannot change society's bias alone. This means that they lack control of the situation and engaging in emotional regulation strategies will likely be more harmful than helpful, as they will take on the emotional burden of the oppressive situation without being able to change it. To better understand this phenomenon, we will explore two situations of discrimination faced by healthcare workers and the harms that come from emotional regulation. Table 1 summarizes the discussion to follow.

**Table 1:** Types of discrimination toward healthcare providers, emotional regulation strategies, and their affective harms.

Type of discrimination	Social and ethical expectations to suppress emotions	Emotional regulation strategies	Affective harms
Coming from patients toward healthcare providers	Duty to care requires emotional management strategies to care for difficult patients	Situational	Feeling isolated within the team; demotivation at work
		Cognitive reevaluation	Sympathy toward the aggressor (the patient or family), causing sadness and confusion
Between colleagues	Professional workplace relationships should be “objective” and “professional” and thus emotions have no place	Attentional, cognitive reevaluation, and emotional suppression strategies	Toxic workplace environment; problems of interprofessional coordination and motivation, affecting care quality

## 2 Case Study 1: Healthcare Providers Facing Discriminatory Patients and the Duty to Care

This first case study will look at what happens when healthcare providers face discriminatory patients or families. Here we will discuss the dilemma between the ethical principle of the duty to care and the need to address discrimination. Duty to care emphasizes the professional obligation to provide care for all patients, even when it is inconvenient or there is a risk to healthcare providers. While the term “duty of care” comes from law, the underlying idea that physicians owe obligations to patients is deeply embedded in medical ethics and is coherent with the principles of beneficence (acting in the patient's best interest) and non‐maleficence (avoiding harm). The expectation that healthcare providers must prioritize their patients leads to the expectation that healthcare providers need to put aside their feelings about the situation/patient. When faced with distressing emotions—be it anger, fear, or disgust—they use emotional regulation strategies to be able to continue care. However, as we will show, when professionals are unable to show justified anger when faced with discrimination, or to find other ways to deal with these feelings in their institutions, this may emotionally burden them as well as influence care quality.

While the duty to care is generally prioritized in healthcare planning and delivery, it is being increasingly recognized that healthcare providers have a right to protect their own physical and mental health and preserve their capabilities to care for current and future patients [12]. It is generally believed that a duty to care exists until the risk of harm is unreasonable; however, the criteria of reasonableness is not clearly defined in scientific literature or professional guidance, creating considerable confusion and variation among structures, as well in individual situations [13]. At the current time, there is no clear guidance on the criteria of *reasonableness* in cases in which healthcare providers experience discrimination and whether healthcare providers can refuse to treat discriminatory patients. This puts healthcare providers facing discrimination into a difficult situation, in particular when they are unsupported by their hierarchies. In addition, it should be recognized that this lack of institutional support is not (always) intentional, in particular in light of busy hospital scheduling and limited human resources; however, it does often lead to prioritizing the discriminatory patient over the discriminated healthcare professional. For instance, a common example is a patient requesting a change of a healthcare provider as they believe the person is not competent or they simply do not wish to be treated by them. While ceding to this request is a practical means of avoiding conflict with the patient, it does little to address the unjust situation or its negative effect on the healthcare provider.

To better understand this phenomenon, we will discuss a qualitative study of ethnic minority healthcare workers and their experiences of both overt and covert racism from patients [14]. This study will show the ways in which patients discriminated toward staff, the reactions of their hierarchies, and the affective harms that emerged. First of all, the study's researchers found several explicit and implicit ways in which patients discriminated toward healthcare staff. This included discounting ethnic minority staff or those who spoke with a foreign accent as “unqualified”; refusing care or asking for another healthcare provider without giving their reasons; or clearly using racist terminology or other remarks to demonstrate their discriminatory views. In these situations, the duty to care was prioritized by *both* the healthcare providers experiencing discrimination and their hierarchies. As one interview participant stated it, *we have been brainwashed somehow. Because every time, we are reminded during all the years of education that the patient comes first. And you have to be understanding and supporting, which is a part of being a doctor. And if something happens, and the patients say anything, you should never take it personally.* As we can see from this quotation, while this healthcare provider regularly has to deal with both direct and indirect discriminatory behavior, the person choses to be “understanding and supportive” rather than to express justified anger or refuse to treat them. In return, this expectation was reinforced by their hierarchies who explicitly prioritized the duty to care over the well‐being of their employees. One interviewee related that her supervisor told her, *you know, we face a lot of people like this every day. So, you should never take it personally. Just put it behind you and just keep going, because at the end of the day if you are going to take everything personally, you are going to feel very bad about it.* From this quotation, we can understand that the supervisor was trying to help the employee to find coping mechanisms to deal with difficult patients. However, by telling her “not to take it personally,” the supervisor gave little acknowledgement of having heard or understood the person's feelings, or even of taking seriously the discrimination. Furthermore, the expectation not to “take everything personally,” puts all of the responsibility on the person suffering from discrimination. Practically, this means that the person being oppressed is not heard or understood, which may leave them feeling sad, angry, and isolated in the team.

In their study, the expectation of healthcare professionals to “stay calm,” “not to take the situation seriously,” or to “put the patient first,” led to several emotional regulation strategies, in particular situational and cognitive reevaluation strategies. For instance, when faced with a discriminatory situation, one response was to avoid finding oneself in the oppressive situation (situational strategy). It was for this reason that some healthcare providers readily accepted the suggestion that another healthcare provider be found when faced with a discriminatory patient who didn't want to be treated by them. While this fixed the problem in the short‐term, when they faced the situation on a regular basis, this led to long‐term repercussions: some decided to quit their jobs or to leave for another service or hospital or decided to work somewhere with a longer commute time or less possibilities of advancement or training. While the conflictual situation was resolved (the person facing discrimination no longer had to deal with their oppressor), it had important social and professional consequences for them and their institutions. They were not able to confront their oppressors or seek redress within their institutions, as the case was swept under the rug; in return, the service/hospital lost a valuable employee and did not address the toxic workplace created due to discrimination.

The second emotional regulation strategy used by healthcare providers was cognitive, including the technique of reappraisal of the situation by “focusing on the duty to care” in order to deflect their attention so that they would not react in anger. An example of cognitive reappraisal in Alberg's study was, for one professional, to find a way to empathize with the patient. Faced with an elderly person who used an explicit racial slur, the professional excused the person by saying that the person had “only seen Africa on television” and thus could not be blamed for their ignorance. This cognitive reappraisal strategy led the professional to develop empathy in order to continue care. While this permitted the person to fulfill their professional duty, it also meant their affects (and the causes of them) were dismissed. This strategy in turn negatively affected their well‐being: while the person said she was not angry toward the patient, she could not do away with her sadness toward the experience. Considering that the workplace did not address the problem, the healthcare professional was left alone with these feelings and received no institutional support. Indeed, in their study, the researchers faced considerable obstacles in participant recruitment due to healthcare providers' fear of jeopardizing their employment or risking relationships with colleagues by speaking out on discrimination.

## 3 Case Study 2: Healthcare Providers Facing Discriminatory Colleagues

In this second debate, we will look at what happens when healthcare providers face discriminatory colleagues. Here we are referring to both explicit discriminatory remarks made by colleagues/hierarchies but also more subtle experiences of institutional discrimination, such as being ignored for promotion or over‐disciplined by managers, which are difficult to address as they remain hidden and based upon unconscious bias. As we will see, these situations also lead to harmful emotional regulation strategies which affect the well‐being of healthcare providers.

To better understand this phenomenon, let us look at the results of a 900+ person study of Black, ethnic minority, and migrant healthcare staff in the United Kingdom on discrimination in the workplace [15]. The study showed that around 44% of respondents experienced discrimination, bullying, and harassment from colleagues, with a disproportional percentage affecting women, black ethnic groups, and migrants. In addition, those lower in the hierarchy, such as nurses and healthcare assistants, were more likely to experience discrimination than those “higher up” such as doctors. Furthermore, those lower on the hierarchy experienced both vertical discrimination (from hierarchies) and horizontal discrimination (among their own staff group). The study also found a correlation in experiencing discrimination and poorer mental and physical health, with a greater tendency to suffer from anxiety or depression, more days sick leave, and significantly less job satisfaction.

A complementary study by Walsh et al. [16] has showed that this type of discrimination often takes the form of micro‐aggressions. Indeed, while overt discrimination continues in the workplace, it may also take more subtle forms. This may include more verbal and non‐verbal comments, looks, or tones that can be difficult to recognize or challenge. In their study of racial microaggressions of nurses in the United States, participants reported multiple types of micro‐aggressions, including as a recipient, bystander, or source. This ranged from subtle comments to overt discriminatory behavior, such as questions by coworkers about their skills and experience or jokes between colleagues about race. A study by Ogunnowo et al. [17] has also showed the negative effects of micro‐aggressions experienced by healthcare providers to their well‐being, including anxiety‐related trauma symptoms, lower job satisfaction, burn‐out and career perceptions.

While we are not aware of studies specifically focusing on the emotional regulation strategies employed by healthcare providers when dealing with micro‐aggressions, researchers are starting to make the link between emotional regulation, micro‐aggressions, and well‐being. For instance, a study by Sawyer [18] looked at the racial microaggressions of 91 racial minority persons in the United States. Their study found a variety of emotional regulation strategies used to deal with discrimination and showed that when situational strategies (avoiding the person) were unrealistic, other emotional regulation strategies, including attentional, cognitive, and emotional suppression strategies were used, with detrimental effects including increased anxiety, trauma, and depression.

Let us better understand this phenomenon through a theoretical case study. Imagine a situation in which a minority nurse faces discrimination from her supervisor because of her social status (gender and race). Rather than criticizing the nurse directly or questioning her professional competencies, the supervisor couches his discriminatory behavior in jokes about her social status. Her justified emotional response would be to respond in anger and to call out her superior for the discriminatory joke. However, three problems ensue if she decides to do this: (1) overtly calling out her supervisor may cause problems with her hierarchy, in particular if the person does not acknowledge their own bias; (2) the remark is couched as a joke, which means the person risks being seen as “too sensitive” or someone “who can't take a joke”; (3) due to bias and stereotypes, responding in anger may disserve the person as it reinforces stereotypes held by her superior (such as the “angry black woman” trope); (4) there is a perception that expressing emotions is unprofessional in healthcare; thus her justified anger may be misinterpreted.

In this situation, the nurse may have no choice but to respond – at least in the short‐term – with an emotional regulation strategy that will suppress or calm her anger to avoid conflict, in particular as she has to continue working (at least in the short‐term) with her supervisor. One emotional regulation strategy may be to put her attention elsewhere, such as not responding to their colleague's joke by looking away (attentional strategy). While this non‐verbal clue could help her supervisor to understand that the joke was inappropriate, we cannot guarantee this sensitivity, particularly in a group setting. For the nurse, just looking away, or focusing on something else, may help her in the short‐term, but she will still be left with her feelings of anger, frustration, and sadness. In the long‐run, this may cause her anxiety, isolation, and a loss of motivation, especially if such jokes are likely to reoccur, creating a toxic work environment for her (and most likely others around her).

A second strategy that she may use is a cognitive reappraisal strategy, such as trying to sympathize with her supervisor, as we saw in the example of patient discrimination (the person responding by saying the patient had only seen “Africa on television”). The nurse may try to take a wider perspective by thinking that her supervisor was trying to ease the tension in the room with a joke; try to justify the behavior by thinking that they were having a bad day; or simply reasoning that their supervisor did not have enough life experience to know better. While this may help them to emphasize with the person, and even try to help them (such as being particularly nice to them when they are having a bad day), the emotional toll falls on the nurse, as they are excusing away the person discriminating them and their legitimate anger about the unjust situation.

A final strategy that the nurse may use is an emotional suppression strategy, such as purposely suppressing their facial muscles to avoid expressing anger or even trying to laugh and joke with their supervisor in return. Again, this strategy does little to deal with the conflictual situation, and may cause considerable anxiety and stress, as the person is forcibly adapting their behavior to the oppressor's expectations. As we can see from this example, inter‐colleague discrimination causes serious affective harms for the healthcare provider, and emotional regulation strategies do not help (but rather harm) them. It also influences their feelings of belonging in the team and the maintenance of interprofessional relationships that are important to care quality.

## 4 Affective Resources and Opportunities for Healthcare Providers

Having described the affective harms for healthcare providers that come from dealing with discriminatory practices by patients and colleagues, it will now be important to turn to what kind of resources and opportunities may be offered to help remedy these harms. Gallegos [19] has proposed that we will move toward affective justice when individuals or groups acquire and can use the affective goods they are owed. He categorizes these affective goods as:

- *Affective resources and opportunities*: activities, spaces, and circumstances that may contribute positively to one's subjective well‐being (including providing self‐care and safe spaces).
- *Affective freedoms*: freedom from interference in the pursuit of subjective well‐being (such as avoiding emotional distress).
- *Affective recognition*: respectful consideration of, and responsiveness to, one's particular needs (and a recognition that one's affects are legitimate)

While studies on discrimination (and in particular on micro‐aggressions) have identified a number of promising strategies to overcome trauma and distress [20], it will be important to specify the kind of affective goods needed in the context of healthcare because affects are governed by social norms in healthcare institutions that generally see their expression as unprofessional; the hierarchical nature of the hospital environment which makes it difficult for persons in lower levels of the hierarchy to do something about their discrimination; and the difficulties coming from the principle of the duty to care toward patients.

1. *Affective resources and opportunities*
  In this first section, we will look at Gallegos' proposal to provide greater affective resources and opportunities for healthcare providers. While such kinds of goods may benefit all healthcare providers, specific methodologies may also need to be developed for those suffering from discrimination. Duker et al. [11] have suggested that redemption methodologies may be used to deal with trauma from discrimination. This type of method involves the construction of new narratives which involve reimaging of events from the past to help understand and inform the future, such as being able to overcome adversity. This may also help some persons to speak out the next time they experience (or witness) discrimination, as they have imagined an alternative. The challenge of using this method in healthcare is that places the responsibility on the person being discriminated, with the expectation that they will become empowered to change the situation. This may not always be possible in situations of hierarchy or the most subtle forms of discrimination, such as the joking behavior we saw in the second case study. However, the advantage of this strategy is that it may help the individual work through emotionally distressing experiences and to be heard and understood in the hospital space. However, due to their limits, such methodologies will need to be combined with other strategies to create a safer work environment.
2. *Affective freedoms*
  Because of the limitations of individual resources, let us now explore how to create a safer work environment through Gallegos' proposition of affective freedoms, in particular his proposition that persons should be freed from circumstances that give rise to emotional distress. However, he nuances this claim by stating, “presumably not all circumstances that give rise to unpleasant emotions, or any instances in which a person's emotional expressions are socially sanctioned, constitute significant harms to that person's subjective well‐being.” Indeed, it is be recognized that healthcare workers face a variety of unpleasant emotions in healthcare work, given that the profession is centered on distressing events and circumstances (diagnosis of a condition, bad prognosis, announcing sudden or coming death, etc.). The emotions lived in these encounters are an inevitable part of healthcare work, and healthcare workers in their mission to treat their patient also accept these occupational risks. However, what we take from Gallegos' proposal is that institutions may work to reduce the emotional distress of those facing discrimination, as these situations tend to exacerbate what is already an emotionally challenging workplace and cause affective harms for those facing it. We therefore suggest that explicit non‐discrimination strategies will need to be employed to strike a balance and ensure that healthcare providers' experiences of discrimination are taken seriously. This can be reinforced with key institutional resource persons, such as affirmative action officers, who can ensure that complaints from healthcare staff are handled according to the hospital's anti‐discrimination policy and legal requirements. The British Medical Association [21] for instance has proposed the following strategies:
  1. Making explicit that discriminatory behavior is not tolerated.
  2. Putting into place confidential reporting mechanisms (whether between colleagues or with patients).
  3. Providing bystanding training to be able to respond even when not directly affected.
  4. Providing emotional support mechanisms for those experiencing discrimination.
  As we can see, if such a global strategy is put into place, it may provide the kind of affective freedoms Gallegos is talking about. By explicitly saying that discriminatory behavior is not tolerated by the institution, both healthcare workers and their hierarchies will be encouraged to respond to discriminatory patients by making clear that they will need to adopt a respectful attitude toward healthcare providers, in spite of their personal opinions and beliefs. Furthermore, training bystanders to respond may facilitate this change, as it will encourage others to defend their colleagues, even if they are not directly affected by discrimination. Finally, providing both a forum for confidential reporting and emotional support mechanisms to talk through their experiences (such as the redemption methodologies described above) may help create an environment in which the person's affective experiences are better heard and understood.
  Another strategy is the creation of mediation spaces in the hospital. While mediation spaces were originally created to allow patients to make complaints about the care they received or conflicts with healthcare providers, they are also used by healthcare providers when dealing with communicational or relational difficulties with patients [22]. While there is currently no research evidence that mediation spaces may be a fruitful resource for those facing discrimination, their methodology suggests they could be. This is because they provide a third party (mediator) who may give information/advice to help resolve conflicts between patients and healthcare providers. When this “safe person” upholds the non‐discriminatory policy in hospital (thus providing an influential support person/bystander), it may help the healthcare provider to be better heard and understood. Secondly, these spaces also address the emotional aspects of the conflictual situation, providing a forum for healthcare providers' affects to be seen, heard, and understood in the hospital space. Furthermore, when data from mediation spaces is integrated into hospital decision making and information gathering, they may create greater awareness of discrimination in the hospital (hopefully) leading to concrete steps to deal with it. The limitation with this resource is that those facing discrimination must feel comfortable using it, in particular due to their need for anonymity to avoid repercussions by colleagues. It may therefore be more useful for cases of patient‐healthcare provider discrimination rather than between colleagues.
3. *Affective recognition*
  Finally, Gallegos proposes that in order to achieve affective justice, we will also need to recognize one's affects as legitimate. Indeed, one of the reasons that healthcare providers facing discrimination find it so difficult to be heard and understood about their justified anger, sadness, or frustration is the social norm that sees emotions are unprofessional/incompatible with healthcare work. Because of this, those who suffer from affective injustice due to their social status (such as the angry black woman trope) will be further sidelined or misunderstood when they express their anger about discrimination. Therefore, a final step for institutions is to create a safer affective space for affects to be recognized as important to care work. We are not advocating here that affects should always be expressed in the consultation or in an interprofessional meetings, but rather that safe spaces be created to help healthcare workers discuss their emotions in an open way. This will give a safer affective space for those experiencing discrimination, but also other healthcare workers facing emotionally challenging situations in care work. Many of these methodologies already exist and can be adapted for the specific problem of discrimination, including Balint groups (which discuss difficult cases) and narrative medicine workshops (where cases of discrimination may be integrated into readings and discussions). These methodologies are just a first step however to work toward a cultural change in the hospital, one in which affects (and experiences of discrimination) can be heard, understood, and addressed.

## 5 Conclusion

This article showed the relevance of the concept of affective injustice to consider how discrimination affects healthcare provider well‐being, quality of care, and motivation at work. We showed the difficulties of dealing with patient‐healthcare provider discrimination, in particular by exploring the professional obligation of the duty to care and social expectations of healthcare providers to manage their emotions in order to “stay professional.” We also showed the difficulties caused by colleague‐to‐colleague discrimination, particularly in cases of micro‐aggressions, and the harms caused by emotional regulation strategies. We ended this article by proposing some preliminary means to make the workplace a safer space for all, in particular by considering what affective resources, opportunities, and freedoms could be put into place. However, there is a long way to go before affective injustice can be achieved for those suffering from discrimination in the workplace. Given that not taking seriously these emotions not only leads to interpersonal distress, but also lower quality of care work and staff retention, affective injustice is an important public health issue that needs to be taken seriously to ensure quality of care for all.

## Conflicts of Interest

The author declares no conflicts of interest.

## Data Availability

The author has nothing to report.
